# Idiopathic Spontaneous Cervical Epidural Hematoma: A Sudden Neurological Catastrophe

**DOI:** 10.7759/cureus.105648

**Published:** 2026-03-22

**Authors:** Lena DeLorenzo, Danielle Thor, Brittany Kenny, Kelly A Schiers

**Affiliations:** 1 Internal Medicine, Jefferson Health New Jersey, Stratford, USA; 2 Critical Care, Jefferson Health New Jersey, Turnersville, USA

**Keywords:** cerebral vascular accident, epidural hematoma, hemiparesis, neck pain, spontaneous cervical epidural hematoma

## Abstract

Spontaneous cervical epidural hematomas (SCEHs) are unique in both their rarity and devastating associated complications. Through this report, the case of a male in his mid-50s with minimal past medical history who developed an acute onset of neck pain secondary to a SCEH with progressive left-sided paralysis is detailed. In doing so, a discussion about the underlying mechanisms of SCEH, or lack thereof, is provided. Emphasis is placed on the patient's clinical presentation, imaging, neurosurgical intervention, and neurological recovery. It is imperative to identify SCEH and provide emergent intervention in the hopes of obtaining neurologic recovery.

## Introduction

Spontaneous cervical epidural hematoma (SCEH) is a rare clinical entity, accounting for less than 1% of all spinal canal-occupying lesions, characterized by the accumulation of blood within the cervical spinal epidural space [1-2]. It typically presents with acute neck pain, followed by rapidly progressing neurological deficits such as quadriplegia or hemiplegia, at times mimicking a cerebrovascular accident (CVA) [1]. SCEH is often idiopathic and requires urgent diagnosis and surgical intervention to prevent permanent neurological impairment [1].

Although SCEH is reported in the literature, a significant portion of cases are linked to predisposing factors such as cardiovascular disease, coagulopathy, and underlying vascular malformations [2-3]. Other cases theorize that concurrent parathyroid adenomas, malignancies, spinal manipulations, the use of anticoagulants or antiplatelet agents, and/or other traumatic events were the source of their associated SCEHs [4-7]. The current report details a genuinely idiopathic SCEH in a middle-aged male, describing the case from diagnosis through definitive management.

## Case presentation

A male patient in his mid-50s with a past medical history of non-insulin-dependent diabetes mellitus, hypertension, and prior osteomyelitis of the left first metatarsal presented to the emergency department via ambulance with a three-hour history of sudden-onset severe neck pain followed by left-sided upper and lower extremity weakness. Prior to symptom onset, he was in his usual state of health. He denied a history of trauma, recent falls or illnesses, bleeding disorders, vascular anomalies, or chronic medication use, including antiplatelet or anticoagulant agents. Due to financial constraints, he had been unable to obtain his outpatient antihypertensive and antihyperglycemic medications for several months prior to presentation. He denied a history of tobacco or alcohol use, endorsed occasional marijuana use, and reported no other illicit substance use. 

On arrival to the emergency department, his vital signs were notable for a blood pressure of 207/104 mmHg; he was afebrile and normocardic. Initial laboratory studies were significant for an elevated glucose of 278 mg/dL and a C-reactive protein (CRP) of 0.60 mg/dL. Complete blood counts (CBC), basic metabolic panel (BMP), erythrocyte sedimentation rate (ESR), hepatic function panel, prothrombin time (PT), partial thromboplastin time (PTT), and international normalized ratio (INR) were unremarkable. Blood cultures were obtained and remained negative. He was awake, alert, and oriented to person, place, and self. Neurologic examination revealed left-sided upper and lower extremity motor and sensory weakness, with intact motor and sensory function of the right extremities. Cranial nerves II-XII were intact. He endorsed neck pain and exhibited decreased cervical spine range of motion. There were no physical examination findings consistent with meningitis. Cardiopulmonary examination was unremarkable. Bladder scan demonstrated greater than 1,000 mL of retained urine, requiring Foley catheter placement. The chest radiograph was unremarkable.

**Figure 1:**
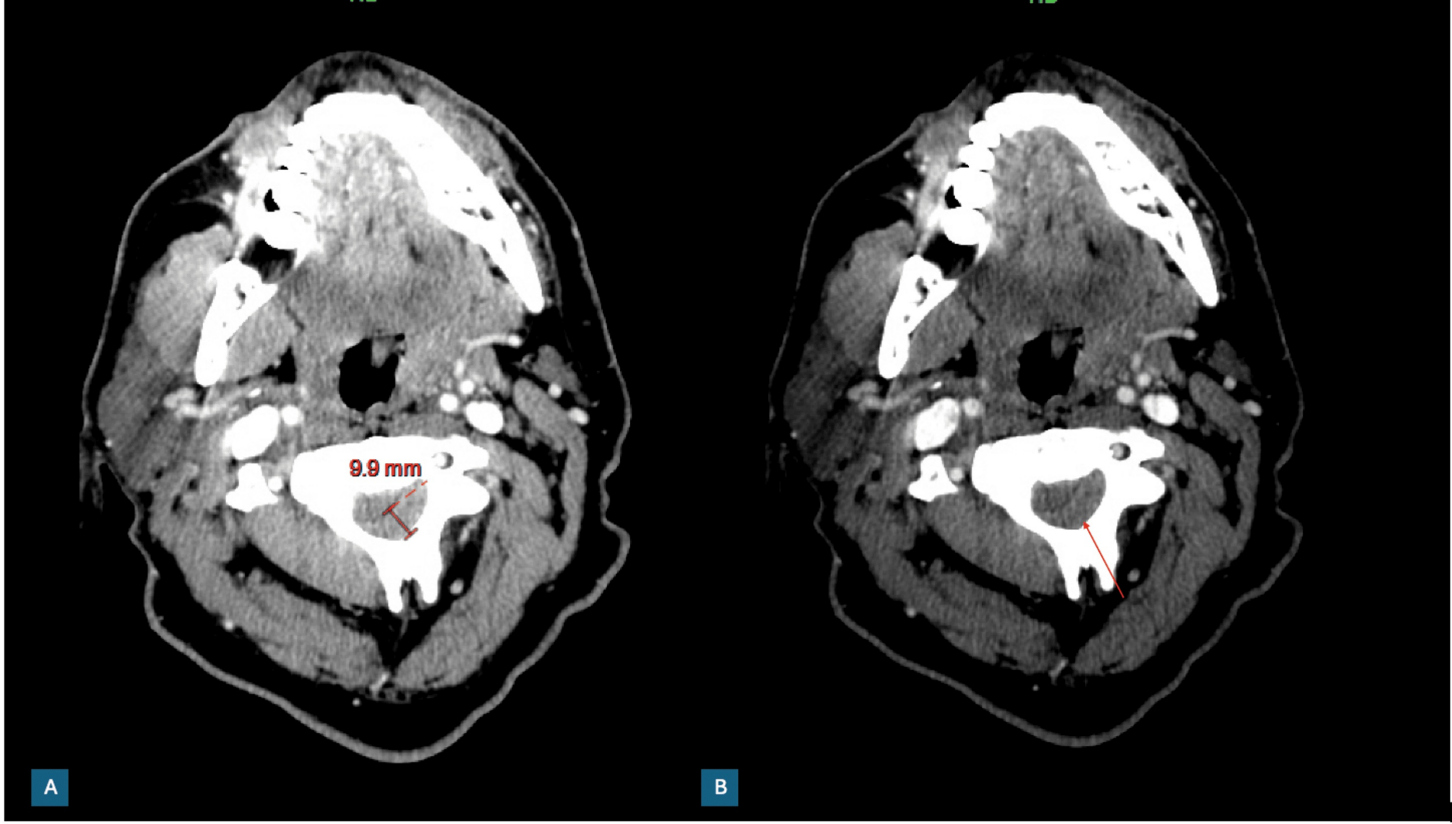
Key image from the emergent CT angiography (CTA) head, demonstrating abnormally high attenuation material in the central canal. (A) Measurement of the high attenuation material up to 1 cm in measurement. (B) An arrow pointing to the area of material.

Shortly after presentation, he developed right-sided upper and lower extremity weakness. Given these new neurologic deficits, a stroke alert was immediately activated. He underwent stat non-contrast computed tomography (CT) of the brain and CT angiography (CTA) of the head and neck, which demonstrated up to 1 cm in thickness of abnormally high-attenuation material in the central canal posteriorly and to the left, causing mass effect at C2-C4 (Figure 1). A teleneurologist was contacted per institutional protocol. The recommendation was to admit the patient to the neurological intensive care unit (ICU), undergo neurosurgical evaluation, and initiate dexamethasone 10 mg daily.

**Figure 2:**
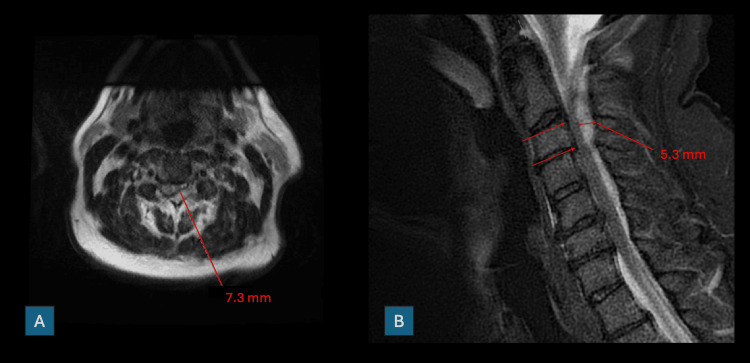
Key images from the emergent MRI brain, which identify the extent of the spontaneous cervical epidural hematoma (SCEH). (A) Axial view. (B) Sagittal view.

Within two hours of admission to the ICU, the patient's neurologic exam deteriorated, progressing to left-sided hemiplegia and right-sided hemiparesis, prompting emergent magnetic resonance imaging (MRI) of the cervical spine. MRI revealed a T1 isointense, partially restricting and non-enhanced collection within the posterior epidural space tracking from the posterior arch of C1 with its thickest component at the level of C2-C3, and trace tracking as far as the level of T2-T3 (Figure 2). These findings were then emergently communicated to the critical care and neurosurgical teams by the neuroradiologist as consistent with an SCEH.

Given these findings, neurosurgery elected to proceed with emergent surgical intervention. The patient underwent hematoma evacuation with posterior cervical laminectomy, medial facetectomy, and foraminotomy from C2-C7, along with posterior cervicothoracic arthrodesis from C2-T1. The procedure was completed without complications. Histopathologic evaluation of the evacuated epidural collection demonstrated blood and fibrin. Immediately postoperatively, the patient’s urinary retention resolved. He regained full function of the right upper and lower extremities, partial motor function and sensation in the left lower extremity; however, motor paralysis of the left upper extremity persisted. On postoperative day 1, he underwent a diagnostic angiogram with the neurosurgical team, which revealed no structural abnormalities. The patient was downgraded from the ICU on postoperative day 2.

During hospitalization, he was evaluated by the hematology/oncology service to assess for underlying coagulopathies that could predispose him to developing a SCEH. Given the normal CBC, coagulation studies, hepatic panel, and renal panel, along with the absence of a personal or family history suggestive of a bleeding disorder, an underlying coagulopathy was considered unlikely, and no further testing was recommended. On postoperative day 9, the patient was discharged to an acute rehabilitation facility. At the time of discharge, neurologic examination demonstrated intact motor function of the right upper and lower extremities, intact sensation in all four extremities, and persistent motor paralysis of the left upper extremity. Over the subsequent three to six months, following extensive physical therapy, the patient was able to regain complete motor function of both the left upper and lower extremities.

## Discussion

SCEH is a rare neurological emergency often misconstrued as a classic ischemic CVA. It is important for clinicians, especially within the neurosurgical and critical care spaces, to maintain a high index of suspicion for SCEH, given the lack of clear etiologies for triggering events. Prompt identification and neurosurgical intervention remain key to mitigating long-term sequelae and improving overall survival [8-10].

While there is no standard workup for SCEH, early imaging, such as a CT head to rule out acute intracranial pathology, followed by MRI brain and cervical spine, is critical. MRI remains the preferred imaging modality for diagnosis, coupled with a detailed neurologic assessment [11-12]. Underlying hematologic conditions should be ruled out during initial evaluation as common risk factors for SCEH, including underlying coagulopathies, as well as vascular anomalies and anticoagulation use [11]. Current literature acknowledges both venous and arterial origins for SCEH, with the most widely accepted theories supporting venous sources [13]. Vascular anomalies can be ruled out via cerebral angiograms and diagnostic imaging such as CTA [11]. In the case of our mid-50-year-old male, a thorough investigation for an underlying source was completed, including a thorough history and physical, and a multidisciplinary review of his case. Multiple conversations were held with the patient and his family members to confirm no history of cervical trauma or other relevant inciting events.

Given the patient’s financial constraints leading to uncontrolled hypertension and hyperglycemia at the time of admission, one could argue that the combination of these uncontrolled conditions may have contributed to the development of his SCEH. The hypertensive component is particularly compelling given the likely effects of sustained, critically elevated blood pressure over weeks to months prior to presentation. However, the literature remains inconclusive. A study of 199 patients with spontaneous spinal epidural hematoma (SSEH), as well as a large meta-analysis of 741 SSEH cases, found no causative relationship between arterial hypertension and the development of SSEH [3,14]. Conversely, one case report has cited uncontrolled hypertension as a potential risk factor for SCEH, and a meta-analysis of 29 cohort studies identified uncontrolled hypertension as a possible risk factor for SSEH [15-16]. To our knowledge, there are no large-scale studies specifically evaluating the effects of uncontrolled hypertension on the development of SCEHs. Furthermore, hypertension alone does not fully explain the anatomical location of the hematoma, as hypertensive hemorrhages more commonly present as spontaneous intracerebral hemorrhages rather than spinal epidural hematomas. Therefore, we postulate that this presentation most likely represents a truly spontaneous SCEH.

The rarity of this presentation has resulted in a limited body of literature describing its incidence and prognostic factors. In a retrospective study of 104 patients with SSEH, the severity of preoperative neurologic deficits, being female, and a history of anticoagulant use at the time of diagnosis were identified as poor prognostic factors [17]. Additionally, a separate multicenter case-control study determined that older age (greater than 73.9 years of age), longer time of symptom onset to diagnosis, involvement of the thoracic spine, and greater than 24 hours to surgical interventions were all associated with poorer outcomes [18]. In the case of our patient, several factors supported a favorable outcome, including the absence of anticoagulant or antiplatelet use, age less than 73.9, early hematoma evacuation (within 12 hours of symptom onset), and cervical spine localization of the hematoma [19-20]. Further research remains essential to better understand the prognostic factors specifically associated with SCEH and to identify potential opportunities for both primary and secondary prevention.

## Conclusions

Although limited to a single-patient presentation, this case highlights an important stroke mimic and emphasizes the need for prompt diagnostic evaluation when associated non-traumatic neck or back pain are present. The risk factors for this condition are not well-defined; therefore, obtaining a thorough history, performing a physical examination, and conducting early MRI play an important role in differentiating SCEH from acute CVAs. Prompt neurosurgical intervention remains the cornerstone of treatment to reduce long-term neurological deficits.
